# Longitudinal assessment of the CXCL10 blood and urine concentration in kidney transplant recipients with BK polyomavirus replication—a retrospective study

**DOI:** 10.1111/tri.13584

**Published:** 2020-02-13

**Authors:** Lukas Weseslindtner, Lea Hedman, Yilin Wang, Robert Strassl, Ilkka Helanterä, Stephan W. Aberle, Gregor Bond, Klaus Hedman

**Affiliations:** ^1^ Department of Virology University of Helsinki Helsinki Finland; ^2^ Center for Virology Medical University of Vienna Vienna Austria; ^3^ Division of Nephrology and Dialysis Department of Medicine III Medical University of Vienna Vienna Austria; ^4^ Division of Nephrology, Transplantation and Liver Surgery University of Helsinki and Helsinki University Hospital Helsinki Finland; ^5^ HUSLAB Helsinki University Hospital Helsinki Finland

**Keywords:** BK virus, CXCL10, kidney transplantation, nephropathy, polyomavirus

## Abstract

In kidney transplant recipients (KTRs), BK polyomavirus (BKPyV) replication may progress to polyomavirus‐associated nephropathy (PVAN). In this retrospective study, we assessed the chemokine CXCL10 in urine and blood samples consecutively acquired from 85 KTRs who displayed different stages of BKPyV replication and eventually developed PVAN. In parallel to progression toward PVAN, CXCL10 gradually increased in blood and urine, from baseline (prior to virus replication) to BKPyV DNAuria (median increase in blood: 42.15 pg/ml, *P* = 0.0156), from mere DNAuria to low‐ and high‐level BKPyV DNAemia (median increase: 52.60 and 87.26 pg/ml, *P* = 0.0010 and *P* = 0.0002, respectively) and peaked with histologically confirmed PVAN (median increase: 145.00 pg/ml, *P* < 0.0001). CXCL10 blood and urine levels significantly differed among KTRs with respect to simultaneous presence of human cytomegalovirus (*P* < 0.001) as well as in relation to the clinical severity of respective BKPyV DNAemia episodes (*P* = 0.0195). CXCL‐10 concentrations were particularly lower in KTRs in whom BKPyV DNAemia remained without clinical evidence for PVAN, as compared to individuals who displayed high decoy cell levels, decreased renal function and/or biopsy‐proven PVAN (median blood concentration: 266.97 vs. 426.42 pg/ml, *P* = 0.0282). In conclusion, in KTRs CXCL10 rises in parallel to BKPyV replication and correlates with the gradual development of PVAN.

## Introduction

BK virus (BKPyV) and JC virus (JCPyV), two human polyomaviruses, cause persistent infection, with the urinary tract as most relevant latency site 1. Since cellular immunity plays a key role in control of these viruses, immunosuppressive treatment after kidney transplantation (KTX) may lead to their reactivation, usually first indicated by asymptomatic viruria 2, 3, 4, 5, 6, 7, 8. Especially when viruria is sustained in the long term, it may be followed by viremia, and progression of viral replication is subsequently indicated by occurrence of high viral loads in urine and blood, which may finally result in significant inflammation, parenchymal injury, and symptomatic disease, termed as polyomavirus‐associated nephropathy (PVAN) 2, 3, 4, 9, 10, 11, 12.

Although BKPyV is the main causative agent, JCPyV may also trigger PVAN 5, 13. Quantitative measurement of BKPyV with or without JCPyV DNA in urine and blood is therefore commonly undertaken during the post‐transplant surveillance 2, 3, 14, 15. Additional correlates of clinical progression are emergence of decoy cells in the urinary sediment, an indicator for cytopathic effects due to urothelial cell infection, the detection of viral aggregates by the Haufen test, BKPyV VP1 mRNA as well as histological evidence of viral inclusions, tubular injury, and inflammatory infiltrates 2, 3, 4, 16, 17, 18.

Host factors, as well as viral determinants, account for the complex pathogenesis of PVAN 3, 4. Immune responses, which on one hand, are essential to control viral replication may on the other hand mediate renal injury. In this process, like in all inflammatory processes, chemokines, structurally related, small chemotactic cytokines orchestrate cell–cell signaling and immune cell trafficking 19, 20. Since chemokines shape immune responses against specific pathogens by recruiting particular subsets of leukocytes to the infection site, they play an essential role and may be used as inflammatory biomarkers in viral diseases 21, 22, 23.

In this study, we longitudinally assessed levels of CXC‐ligand 10 (CXCL10), a chemokine which attracts and activates immune cells with a cytokine profile of T‐helper cells Type 1 (Th1), aiming to determine whether CXCL10 correlates with an increase and clinical progression of BKPyV replication 24, 25, 26. Our analysis also included the chemokines CCL8, CXCL16, and CCL20, candidate markers for outcome and severity of human cytomegalovirus (HCMV) infections in solid organ recipients 22, 23, 27.

## Materials and methods

### Patients and sample acquisition

This retrospective study included pairs of blood and urine samples from a total of 95 KTRs (35 female, 60 male) including controls, who underwent KTX between March 2008 and September 2014. All KTRs underwent virological routine testing which consisted of PCR analyses for BKPyV and JCPyV DNA in urine and blood, collected pairwise on the same day of the post‐transplant follow‐up, respectively, and HCMV DNA in blood. Eighty‐five patients were selected for the study by retrospective review of virological test results and were included when at least one episode of BKPyV DNAemia with DNA loads ≥1000 copies/ml occurred during the post‐transplant follow‐up. Ten KTRs, who underwent transplantation and post‐transplant surveillance during the same time period, served as controls since they lacked any evidence of BKPyV, JCPyV, or HCMV replication (see Table S1). Further information on the immunosuppressive treatment, the schedule of sample acquisition and virological routine testing as well as on the storage of samples is given in Supplemental Material and Methods. Sample collection and storage as well as the whole study protocol was approved by the local ethics committee of Medical University of Vienna, which concluded that no written informed consent from the patients was required (EK1035/2016, EK2064/2016). The study was performed in accordance with the Helsinki Declaration.

### Quantitative PCR

Information on the protocols used for quantitative BKPyV, JCPyV, and HCMV PCR analyses is given in Appendix S1.

### Criteria for grouping patients and selection of samples

Out of the 85 KTRs, who were included in the study because they displayed at least one episode of BKPyV DNAemia, 56 displayed BKPyV DNAemia in the absence of significant JCPyV and HCMV DNAemia. Thirteen KTRs simultaneously developed BKPyV and JCPyV DNAemia (>1000 copies/ml, respectively) and 16 simultaneously developed BKPyV and HCMV DNAemia. Among these groups, there was no significant difference with respect to gender, age, transplant type, donor characteristics, cold ischemia time, delayed graft function, HLA‐mismatches, AB0 and HLA‐incompatibilities, duration of follow‐up and the median time point BKPyV DNAemia occurred (Table S1).

To assess individual CXCL‐10 kinetics during progression of BKPyV replication, we retrieved and analyzed 148 specific blood/urine sample pairs, collected from the 56 KTRs with mere BKPyV HCMV DNAemia due to routine testing. These samples specifically fulfilled the inclusion requirement that they had been collected during different stages of BKPyV replication (mean number of sample pairs per patient: 2.64, range: 2–4). As shown in Table 1, these stages were defined as: (i) baseline (before BKPyV detection, with undetectable BKPyV, JCPyV, and HCMV DNA in urine and blood, no evidence for any other infection nor rejection, normal levels of C‐reactive protein and leukocytes, normal urinary cytology, no drop in renal function), (ii) BKPyV detection only in urine (BKPyV DNAuria) but not in blood, (iii) BKPyV DNAuria plus low‐level BKPyV DNAemia (blood DNA levels <1000 copies/ml), (iv) BKPyV DNAuria plus BKPyV DNAemia (blood DNA levels ≥1000 copies/ml) but no significant decoy cell levels, (v) BKPyV DNAemia (>1000 copies/ml) together with detection of decoy cell levels >20%, a decrease of eGFR ± histological evidence for PVAN. The mean sampling intervals are shown in Table 1. When multiple samples were acquired from one patient during the same stage of BKPyV replication, only the initial sample that fulfilled the respective criteria was selected for the study.

**Table 1:** Criteria for grouping patients and samples acquired during progression of BKPyV replication

Patient group	BKPyV DNAemia						BKPyV & JCPyV DNAemia	BKPyV & HCMV DNAemia	Controls
Number of patients (*n*)	56						13	16	10
Stages of BKPyV replication		Baseline	BKPyV DNAuria, no DNAemia	Low level BKPyV DNAemia (<10^3^ copies/ml)	BKPyV DNAemia (≥10^3^ copies/ml)		BKPyV & JCPyV DNAemia (≥10^3^ copies/ml respectively)	BKPyV & HCMV DNAemia	–
					Decoy cells <20%	Decoy cells >20%			
BKPyV	In urine*	Undetectable	1.8 × 10^5^, 2.7 × 10^2^–1.5 × 10^11^	1.1 × 10^6^, 5.5 × 10^3^–8.1 × 10^9^	2.5 × 10^8^, 2.6 × 10^4^–5.4 × 10^10^	4.2 × 10^9^, 1.9 × 10^4^–4.3 × 10^11^	5.3 × 10^9^, 1.1 × 10^7^–1.9 × 10^11^	2.4 × 10^9^, 8.2 × 10^3^–8.9 × 10^10^	Undetectable
	In blood*	Undetectable	Undetectable	4.5 × 10^2^, 1.0 × 10^2^–9.6 × 10^2^	4.4 × 10^3^, 1.0 × 10^3^–1.9 × 10^5^	5.2 × 10^4^, 1.7 × 10^3^–7.4 × 10^6^	3.8 × 10^4^, 6.6 × 10^3^–4.2 × 10^8^	5.4 × 10^4^, 7.4 × 10^2^–3.8 × 10^6^	Undetectable
JCPyV	In blood*	Undetectable	Undetectable	Undetectable	Undetectable: 28/31 Detectable: 3/31	Undetectable: 40/47 Detectable: 7/47	Detectable: 13/13	Undetectable: 11/16 Detectable: 5/16	Undetectable
	In blood*	Undetectable	Undetectable	Undetectable	2.1 × 10^2^, 1.5 × 10^2^–2.5 × 10^2^	7.8 × 10^1^, 2.8 × 10^2^–7.1 × 10^2^	4.9 × 10^3^, 1.6 × 10^3^–2.7 × 10^5^	3.6 × 10^4^, 4.5 × 10^2^–1.7 × 10^5^	Undetectable
HCMV in blood*		Undetectable	Undetectable	Undetectable	undetectable	Undetectable	Undetectable	9.6 × 10^2^, 2.0 × 10^2^–4.7 × 10^4^	Undetectable
Decoy cells in urinary sediment (>20%)		Undetectable	Undetectable	Undetectable	No	47/47	13/13	7/16	Undetectable
Decrease of eGFR†		No	No	No	No	23/47	6/13	4/16	No
PVAN verified by histology‡		No	No	No	No	15/47	5/13	2/16	No
Number of urine/serum sample pairs§		16	33	21	31	47	13	16	10
Interval between sample acquisition¶		70, 20–265		184, 56–512		177, 56–770		136, 41–581	

BKPyV, BK Polyomavirus; JCPyV, JC Polyomavirus; HCMV, Human Cytomegalovirus; eGFR, estimated glomerular filtration rate; PVAN, Polyomavirus associated nephropathy.

* Median DNA load, range; copies/ml.

^†^ ≥15% decrease of eGFR, as compared to mean of the 3 preceding measurements.

^‡^ Biopsy within 1 week before/after sample acquisition.

^§^ One pair per patient and stage respectively.

^¶^ Median days, range.

In order to compare chemokine levels among individual patients, the 56 KTRs with mere BKPyV DNAemia were sub‐grouped based on the clinical severity of the respective BKPyV DNAemia episode. Although their BKPyV DNA loads exceeded 1000 copies/ml, 9 of the 56 KTRs displayed no significant decoy cell levels (<5%) in urinary sediment during any BKPyV DNAemia episode, renal function remained stable and no biopsy was therefore performed during the entire follow‐up. The other 47 KTRs, in contrast, displayed at least one BKPyV DNAemia episode in which significant decoy cell levels (defined as >20%) were concomitantly detected. Of those 47, 23 (48.9%) additionally showed a ≥15% decrease of estimated glomerular filtration rate (eGFR), as compared to the mean of three measurements which immediately preceded the initial occurrence of the respective high‐level DNAemia episode (median interval: 41 days, range: 13–98). In 15 patients, PVAN was confirmed with a renal biopsy obtained within a week before or after blood/urine sampling. In the remaining KTRs, no biopsies were performed within this time period.

In addition to the 47 patients with mere BKPyV DNAemia, all 13 KTRs, who simultaneously displayed high‐level BKPyV and JCPyV DNAemia, showed decoy cells >20%. An eGFR decrease occurred in 6 (46.2%) and PVAN was histologically confirmed in 5 of these 13 KTRs.

Histological confirmation of PVAN was based on the detection of injured tubules with or without interstitial nephritis, viral inclusion bodies, and/or positive SV40 T‐Antigen staining 28. In all biopsies with confirmed PVAN, pathologists specifically described no histological evidence for rejection 29. However, 10 KTRs with high‐level BKPyV DNAemia subsequently developed allograft rejection during the same BKPyV DNAemia episode, which was confirmed by histology using Banff criteria (borderline T‐cell‐mediated rejection: *n* = 7, CD4d+ rejection: *n* = 1, acute cellular rejection: *n* = 1, T‐cell‐mediated rejection 2a: *n* = 1) 29.

As described, urine/blood sample pairs from 10 KTRs who did not display any BKPyV, JCPyV, and HCMV replication were included as controls, and samples from these controls fulfilled the same criteria as baseline samples (C‐reactive protein and leukocytes in normal range, normal urinary cytology, and no eGFR decrease).

### Quantification of CXCL10, CCL8, CXCL16, and CCL20

CXCL10, CCL8, CXCL16, and CCL20 were quantified by a commercially available ELISA test and LuminexTM xMAP technology 30. More information is given in Appendix S1.

### Statistical methods

Within‐patient comparisons of CXCL‐10 in blood and urine were performed using paired nonparametric t‐tests (Wilcoxon matched‐pairs signed‐rank test). Differences in CXCL10, CCL8, CCL20, and CXCL16 concentrations (including blood/urine ratios) between KTRs with BKPyV DNAemia with and without progression to PVAN as well as among patients with additional JCPyV, HCMV DNAemia were compared by unpaired nonparametric Kruskal–Wallis and Dunn´s multiple comparison tests. CXCL10 between baseline samples and control individuals was compared by Mann–Whitney *t*‐test. Effect sizes are reported as *d*_Cohen_. Receiver operating characteristic (ROC) analyses were performed to analyze the association of CXCL10 with high‐level BKPyV DNAemia, clinical manifestations, and/or histological evidence of PVAN. For all statistical tests, a two‐tailed *P*‐value of <0.05 was considered statistically significant. graphpad prism version 8.0 software was used for statistical analyses.

## Results

### CXCL10 in blood and urine during progression of BKPyV replication

First, we analyzed whether progression of BKPyV replication toward DNAemia and PVAN was associated with a response of the inflammatory chemokine CXCL10. Therefore, we quantified CXCL10 in 148 pairs of urine and blood samples acquired from 56 KTRs with BKPyV (but not JCPyV or HCMV) DNAemia at multiple time points and during different stages of BKPyV replication (Table 1).

**Figure 1:**
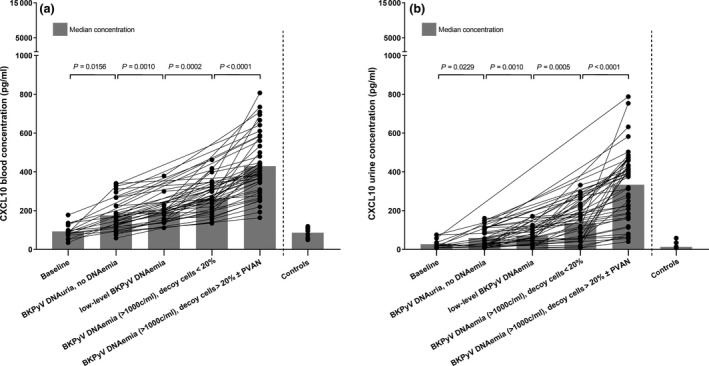
Increase of inflammatory CXCL10 due to progression of BKPyV replication. In 56 KTRs, CXCL10 gradually increases along with progression of BKPyV replication toward PVAN. (a) Blood CXCL10 concentrations increase from baseline (prior to virus replication; *n* = 16 samples) to exclusive BKPyV DNAuria (BKPyV detection only in urine but not in blood, *n* = 33 samples). They further rise when low‐level BKPyV DNAemia additionally emerges (*n* = 21 samples) and even more increase when BKPyV DNA loads subsequently exceed 1000 copies/ml (*n* = 31 samples). CXCL10 in blood finally peaks when BKPyV DNAuria, DNAemia, urinary decoy cells (>20%), an eGFR decrease, and histologically proven PVAN are simultaneously detectable (*n* = 47 samples). (b) Analogously to the blood concentration, CXCL10 in urine increases from baseline to exclusive BKPyV DNAuria, from BKPyV DNAuria to low‐level DNAemia and further to DNaemia ≥ 1000 copies/ml without PVAN. Urinary CXCL10 also peaks when, in addition to BKPyV DNAemia, decoy cells, an eGFR decrease, and histological evidence of PVAN simultaneously emerge. Gray bars indicate median CXCL10 concentrations.

As shown in Fig. 1a and Table S2, from first BKPyV detection in urine toward histologically proven PVAN, we observed a gradual increase of CXCL10 in blood. CXCL10 concentrations significantly increased from baseline to BKPyV DNAuria only (median increase: 42.15 pg/ml, 95% CI: 9.11–151.84, *P* = 0.016). This CXCL10 baseline concentration (before BKPyV detection) did not significantly differ from the one in controls who did not display any BKPyV, JCPyV, or HCMV detection during follow‐up (median difference: −0.81 pg/ml, 95% CI: −32.72 to 21.98, *P* = 0.74). CXCL10 in blood further rose when in addition to mere BKPyV DNAuria low‐level DNAemia emerged (median increase: 52.60 pg/ml, 95% CI: 41.55–78.82, *P* = 0.001) and further increased when low‐level DNAemia progressed to DNA loads ≥1000 copies/ml, but no evidence for PVAN was yet detected (decoy cells <20%, no eGFR decrease; median increase: 87.26 pg/ml, 95% CI: 55.42–153.51, *P* = 0.0002). CXCL10 in blood finally peaked when BKPyV DNAuria and DNAemia concomitantly occurred with clinical manifestations (significant decoy cell levels ± eGFR decrease) and/or histological evidence of PVAN (median increase: 145.00 pg/ml, 95% CI: 120.20–227.83, *P* < 0.0001).

Next, we analyzed whether progression of BKPyV replication toward DNAemia and PVAN was associated with an inflammatory CXCL10 response in urine analogously to blood concentrations. Therefore, we quantified CXCL10 in urine samples obtained pairwise with blood samples and found that CXCL10 urine concentrations also increased due to progression of BKPyV replication. As shown in Fig. 1b and Table S2, CXCL10 in urine rose from baseline to BKPyV DNAuria alone (median increase: 14.15 pg/ml, 95% CI: −1.48 to 38.23, *P* = 0.0223) and increased from this to low‐level DNAemia (median increase: 36.44 pg/ml, 95% CI: 16.59–80.91, *P* = 0.001). The CXCL10 urine concentration further increased from low‐level BKPyV DNAemia when viral DNA loads exceeded 1000 copies/ml but no clinical manifestations of PVAN were observed yet (median increase: 87.59 pg/ml, 95% CI: 39.02–148.07, *P* = 0.0005). CXCL10 in urine finally peaked when, in addition to DNAemia, significant levels of decoy cells, a decrease of eGFR and/or histological evidence for PVAN were detected (median increase: 173.50 pg/ml, 95% CI: 129.27–280.41, *P* < 0.0001).

The individual blood/urine ratio of the CXCL10 concentration did not significantly change during different stages of BKPyV replication until an eGFR decrease occurred and/or biopsy‐proven PVAN developed. As compared to the other stages, evidence for PVAN was associated with a significant decrease of the ratio due to a disproportionately strong increase of urine CXCL‐10 levels (median change of the blood/urine ratio: −0.89, 95% CI: −7.38 to −0.69, *P* = 0.0042).

### CXCL‐10 levels in additional JCPyV, HCMV DNAemia, and rejection

**Figure 2:**
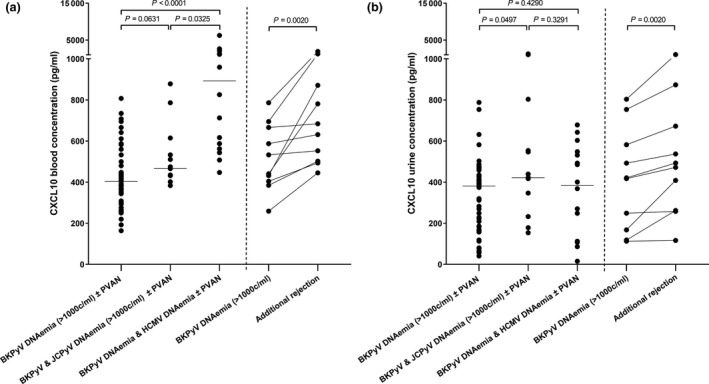
CXCL‐10 levels in additional JCPyV and HCMV DNAemia and rejection. (a) Patients (*n* = 13) who, in addition to BKPyV DNAemia display JCPyV DNAemia (>1000 copies/ml, respectively), show a trend toward higher CXCL10 blood concentrations than those without JCPyV (*n* = 47). The highest CXCL10 blood concentrations are found in KTRs who, in addition to BKPyV DNAemia, show DNAemia of HCMV (*n* = 16). Of note, CXCL‐10 blood concentration significantly increases when 10 KTRs with BKPyV DNAemia subsequently develop additional allograft rejection. (b) KTRs who, in addition to BKPyV display JCPyV DNAemia, also show a slightly higher CXCL10 urine concentration than those without JCPyV DNAemia. The CXCL10 concentration in urine does not significantly differ among KTRs with BKPyV DNAemia (with or without JCPyV DNAemia) and those with additional HCMV DNAemia. Analogously to blood levels, CXCL10 in urine significantly increases when KTRs with BKPyV DNAemia subsequently develop additional allograft rejection.

Then, we compared CXCL10 in blood at BKPyV DNAemia (>1000 copies/ml) with the CXCL‐10 concentration in 13 KTRs who additionally displayed JCPyV DNAemia (>1000 copies/ml), as well as in 16 KTRs with concomitant HCMV DNAemia. Of note, CXCL10 in blood showed a trend for higher concentrations in KTRs with additional JCPyV DNAemia than in patients with mere BKPyV DNAemia (median concentration: 466.03 vs. 404.44 pg/ml, 95% CI: 430.33–613.59 vs. 385.60–473.74, *P* = 0.0631, Fig. 2a). The highest CXCL10 blood concentrations were however encountered in KTRs who, in addition to BKPyV, displayed HCMV DNAemia (median concentration: 892.87 pg/ml, 95% CI: 590.29–2126.10, *P* < 0.0001 vs. BKPyV DNAemia, *P* = 0.0325 vs BKPyV and JCPyV DNAemia, *d*_Cohen_: 1.50, Fig. 2a, Table S2).

Of note, there was a significant increase of the CXCL‐10 blood concentration when 10 KTRs of our study cohort displayed BKPyV DNAemia and subsequently developed additional allograft rejection (median increase: 151.94 pg/ml, 95% CI: 45.47–542.50, *P* = 0.002, Fig. 2a).

Analogously to blood levels, the CXCL‐10 urine concentration was slightly higher in KTRs, who in addition to BKPyV DNAemia displayed JCPyV DNAemia than in KTRs with BKPyV DNAemia alone (median concentration: 422.15 vs. 381.26 pg/ml, 95% CI: 325.48–726.24 vs. 281.15–386.61, *P* = 0.0497, *d*_Cohen_: 0.77). In contrast to blood levels, CXCL10 in urine did not significantly differ between KTRs with BKPyV DNAemia and those who additionally displayed HCMV DNAemia (median concentration: 384.59 pg/ml, 95% CI: 259.27–485.82, *P* = 0.4290 vs. mere BKPyV, *P* = 0.3291 vs BKPyV and JCPyV DNAemia, Fig. 2b). Therefore, a significantly higher CXCL‐10 blood/urine ratio was observed in KTRs with additional HCMV DNAemia, as compared to patients with BKPyV ± JCPyV DNAemia (median ratios: 1.12 for mere BKPyV, 1.10 for BKPyV and JCPyV and 3.00 for additional HCMV DNAemia, *P* = 0.008, *d*_Cohen_: 0.90).

In contrast and analogously to blood levels, there was a significant increase of the CXCL‐10 urine concentration when 10 KTRs with BKPyV DNAemia subsequently developed additional allograft rejection (median increase: 81.22 pg/ml, 95% CI: 39.78–169.11, *P* = 0.0020, Fig. 2b).

### CXCL10 differences in relation to BKPyV‐associated clinical manifestations

Next, we analyzed whether CXCL10 in blood and urine differed among KTRs with respect to the most severe clinical manifestation which concomitantly emerged with BKPyV DNAemia during the follow‐up. Therefore, KTRs with episodes of BKPyV DNAemia were grouped as shown in Table 2. In 20 KTRs, the most severe manifestation of BKPV (±JCPyV) replication was histological evidence of PVAN; in 12 KTRs, in whom no biopsy was performed, the most severe clinical manifestation was a decrease of eGFR. In these patients, the simultaneously detected decoy cell levels were high (>30%), while in 9 KTRs, in contrast, BKPyV DNAemia was associated with no significant levels of decoy cells (<5%) and eGFR remained stable. Although BKPyV DNA loads exceeded 1000 copies/ml in these patients, viral loads were significantly lower than in KTRs with histological and/or clinical evidence of PVAN (Table 2). Absolute eGFR values at the time point of BKPyV DNAemia were also significantly lower in the 20 KTRs with histologically verified PVAN than in the nine patients with low decoy cell levels and lower BKPyV DNA loads (median difference: −11.69 ml/min/1.73, 95% CI: −23.88 to −1.92, *P* = 0.0213, *d*_Cohen_ = 0.96).

**Table 2:** Patient groups based on the clinical severity of BKPyV‐associated manifestation during the follow‐up.

Groups and differentiation criteria	Decoy cells < 5%, no eGFRdrop	eGFR drop	Histologically verified PVAN	Difference among the groups (*P*)
Number of patients (*n*)	9	12	20	
BKPyV DNA in blood (median,range; copies/ml)	2.0 × 10^3^, 1.0 × 10^3^–2.7 × 10^4^	3.2 × 10^4^, 5.3 × 10^3^–1.1 × 10^6^	1.4 × 10^5^, 1.1 × 10^4^–4.2 × 10^8^	<0.0001
Decoy cells in urinary sediment (median, range; %)	1, 0–5	45, 30–95	90, 30–98	<0.0001
Drop of renal function (≥15% decrease of eGFR, as compared to mean of the three preceding measurements)	0/9	12/12	17/20	<0.0001
PVAN verified in histology (biopsy within 1 week before/after sample acquisition)	No biopsy performed	No biopsy performed	20/20	<0.0001

BKPyV, BK polyomavirus; eGFR, estimated glomerular filtration rate; PVAN, polyomavirus‐associated nephropathy.

**Figure 3:**
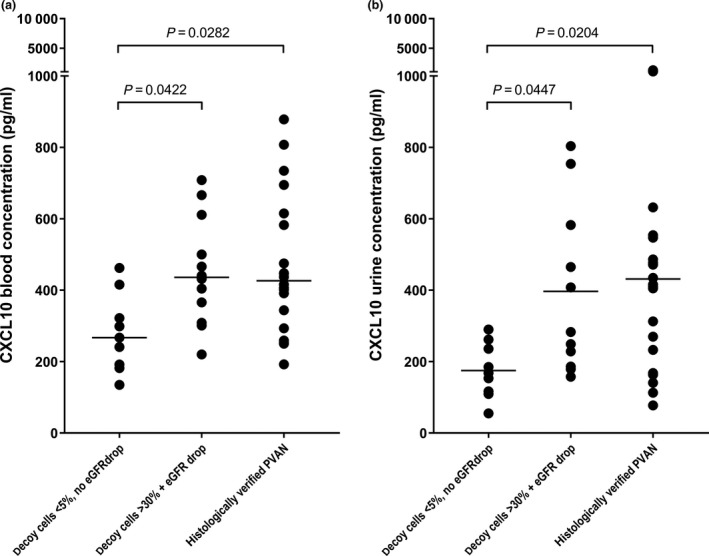
CXCL10 in relation to BKPyV‐associated clinical manifestations. (a) In episodes of significant BKPyV DNAemia (DNA loads exceeding 1000 copies/ml) CXCL10 in blood was levels are significantly lower in KTRs who display lower BKPyV DNA loads, lower decoy cell levels (<5%), and no eGFR decrease (*n* = 9), as compared to KTRs who display a simultaneous eGFR decrease, higher viral loads, and higher decoy cell levels (>30%; *n* = 12), as well as in KTRs in whom PVAN is concomitantly verified by histology (*n* = 20). (b) Also, CXCL10 in urine is significantly higher in KTRs who display an eGFR decrease, higher viral DNA loads, and significant higher levels of decoy cells as well as in KTRs in whom PVAN is histologically verified, as compared to patients with lower BKPyV DNA loads, no significant decoy cell levels and no concurrent eGFR decrease.

As shown in Fig. 3a and Table S2, CXCL10 in blood was significantly lower in KTRs with lower DNA loads, lower decoy cell levels (<5%), and no eGFR decrease, as compared to KTRs with higher viral loads, higher decoy cell levels and a simultaneous decrease of eGFR (median concentration: 266.97 vs. 436.02 pg/ml, 95% CI: 196.09–362.66 vs. 356.77–547.10, *P* = 0.0422). Peak CXCL‐10 blood concentrations, however, were observed in KTRs in whom PVAN was histologically verified (median concentration: 426.42 pg/ml, 95% CI: 375.39–557.33, *P* = 0.0282, *d*_Cohen_ = 0.86).

In addition, CXCL10 in urine was higher during BKPyV DNAemia in KTRs who displayed higher DNA loads, significant levels of decoy cells and an eGFR decrease than in KTRs who did not (median concentration: 345.42 vs. 168.09 pg/ml, 95% CIs: 254.73–538.69 vs. 115.93–234.00, *P* = 0.0447) and peaked in patients in whom PVAN was verified histologically (median concentration: 412.97 pg/ml, 95% CI: 291.90–571.03, *P* = 0.0204, *d*_Cohen_ = 0.885, Fig. 3b).

### Association of CXCL10 with high‐level BKPyV DNAemia and BKPyV‐associated disease

Then, we analyzed CXCL10 levels in high‐level BKPyV DNAemia (presumptive for PVAN) using 10 000 copies/ml (but not decoy cell levels or an eGFR decrease) as reference. Out of the 56 KTRs with peak BKPyV DNA levels >1000 copies/ml, 42 KTRs displayed viral loads >10 000 copies/ml, while 14 patients did not. CXCL10 serum concentrations were significantly higher in these 42 KTRs with peak DNA loads >10 000 copies/ml than in the 14 KTRs with lower viral loads (median CXCL10 concentration: 411.22 vs. 310.15 pg/ml, 95% CI: 438.77–162.82 vs. 318.64–116.81, *P* = 0.0122, *d*_Cohen_ = 0.70). For CXCL10 in urine, however, no statistically significant difference was observed (median concentration: 343.50 vs. 292.91 pg/ml, 95% CI: 258.75–366.52 vs. 194.23–414.98, *P* = 0.7152). As shown in Fig. S1, we also performed ROC analyses to evaluate the association of the CXCL‐10 concentration with manifestations of BKPyV‐associated disease. Of note, the area under the curve (AUC) was slightly higher when clinical manifestations and/or histological evidence PVAN rather than high‐level BKPyV DNAemia >10 000 copies/ml were used as a reference (Table 2; Fig. S1).

### CCL8, CXCL16, and CCL20 blood levels in relation to BKPyV‐associated clinical manifestations

**Figure 4:**
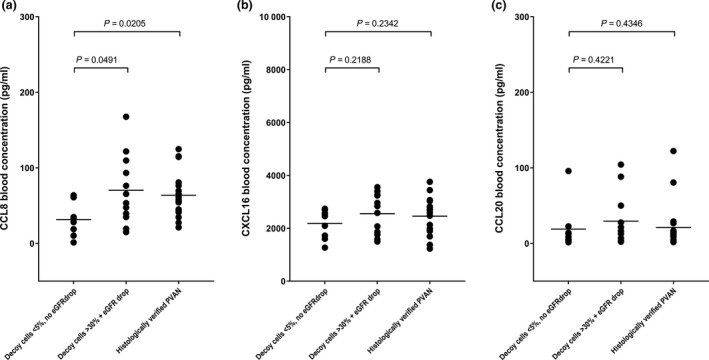
CCL8, CXCL16, and CCL20 in blood in relation to BKPyV‐associated clinical manifestations. (a) CCL8 in blood is significantly higher in KTRs with BKPyV DNAemia (DNA loads exceeding 1000 copies/ml) who display an eGFR decrease, higher viral loads, and higher decoy cell levels (*n* = 12) as well as histologically confirmed PVAN (*n* = 20), as compared to KTRs who display lower BKPyV DNA loads, lower levels of decoy cells, and no concurrent eGFR decrease (*n* = 9). (b) CXCL16 and (c) CCL20 in blood do not significantly differ in KTRs with BKPyV DNAemia in relation to levels of BKPyV DNA, decoy cells, the concurrent presence of an eGFR decrease as well as to histological evidence of PVAN.

Next, we investigated whether concentrations of other T‐cell associated chemokines differed in relation to the severity of BKPyV replication. Therefore, we quantified CCL8, CXCL16, and CCL20 in blood in the groups of KTR as shown in Table 2 and found that CCL8 blood concentrations were significantly higher in KTRs with BKPyV DNAemia who displayed higher viral loads, significant levels of decoy cells, and an eGFR decrease as well as in KTRs who displayed histological evidence of PVAN as compared to KTRs who did not (median concentrations: 59.40 vs. 57.79 vs. 32.34 pg/ml, 95% CIs: 41.27–99.28 vs. 50.49–76.80 vs. 15.54–47.52, *P* = 0.0491 vs. eGFR decrease, *P* = 0.0205 vs. PVAN, *d*_Cohen_: 0.88, Fig. 4a). In contrast, CXCL16 and CCL‐20 in blood did not significantly differ among KTRs with BKPyV DNAemia with respect to the severity of clinical manifestations associated with respective BKPyV DNAemia episodes (Fig. 4b,c).

## Discussion

In this study, we longitudinally analyzed CXCL10 in KTRs during different stages of BKPyV replication and demonstrate that, along with progression of BKPyV replication, CXCL10 in blood and urine gradually increases, from viral DNAuria over low‐ and high‐level DNAemia to a decrease of renal function and histological evidence PVAN. These findings suggest that CXCL10 concentrations in blood and urine might reflect the stepwise increase of inflammation and development of polyomavirus‐associated disease in KTRs, a fact that should specifically be considered when CXCL10 is proposed as particular marker of allograft rejection 25, 31, 32, 33, 34, 35, 36, 37.

Indeed, CXCL10 attracts and activates T‐cells with a Th1 cytokine profile, and since its production can again be induced upon stimulation with Th1‐derived cytokines like interferon‐γ (IFN‐γ), it apparently participates in a positive feedback loop 24, 25. CXCL10 is thus essential to elicit a Th1‐shaped cellular immune response against viruses, but on the other hand, contributes to tissue inflammation and injury 38. Consequently, CXCL10 has been proposed as marker of immune activation, severity, and therapy response in diverse viral diseases but also of allograft rejection 21, 25, 30, 31, 32, 33, 34, 35, 36.

In previous studies, elevated CXCL10 urine concentrations were equally detected when KTRs displayed BKPyV nephritis or DNAemia as well as allograft rejection 33, 34, 39. Elevated blood concentrations were encountered in KTRs with BKPyV detection in urine and/or serum 8, 33, 40. Our present data are in accordance with and extend these former findings, indicating that a CXCL10 increase due to BKPyV can be equally detected in urine and blood, occurs stepwise and in proportion to the extent of viral replication, and correlates with a decrease of renal function and histological verification of PVAN. Of note, a similar observation has been made for Interleukin‐6, which increases with rising BKPyV loads 41.

The gradual CXCL10 response we observed here, not only discloses that intrarenal BKPyV replication and CXCL10 secretion into urine and blood are functionally linked, but suggests its cumulative inflammatory effect on the occurrence of nephropathy. A correlation of urinary CXCL10 levels with tubule‐interstitial inflammation and grade of tubulitis was previously reported, while upregulation of CXCL10 genes could pose the underlying functional background in patients with PVAN 33, 42, 43, 44.

Our particular finding that not only urine but also blood CXCL10 concentrations were elevated over baseline during mere BKPyV DNAuria, furthermore highlights that even early, clinically silent, and locally restricted BKPyV replication in the allograft is sufficient to elicit a systemic CXCL10 rise and trigger inflammation. Since increased CXCL10 levels have been associated with long‐term allograft dysfunction in KTRs, an increased CXCL10 concentration due to low level but sustained BKPyV replication could also possibly adversely influence allograft survival 33, 35, 36, 39, 45, 46. Correspondingly, a constantly low CXCL10 urinary concentration was recently correlated with histologically stable allografts and freedom of rejection 47. Thus, it is indeed possible that CXCL10 might serve as inflammatory marker to individualize immunosuppressive treatment, this, however, requires further studies.

Although we demonstrated that CXCL10 gradually increases along with BKPyV replication, the potential use of CXCL10 as biomarker for PVAN may be limited. Of note, allograft rejection may also be associated with increased CXCL10 levels, whereby multiple studies have proposed CXCL10 as a rejection marker 25, 31, 32, 39, 42, 46. In this regard, our observation that CXCL10 in blood and urine already increased for mere BKPyV DNAuria, particularly suggests that absence of BKPyV should be verified, before a CXCL10 rise can be considered an indicator of allograft rejection. Indeed, we observed that CXCL10 further increased when KTRs with BKPyV DNAemia subsequently developed allograft rejection, but considering that CXCL10 responses might vary among individuals, it seems questionable whether quantitative differences might ultimately discriminate between PVAN and rejection.

With this regard, we also demonstrated that other viral agents influence the CXCL10 concentration in KTRs, and especially HCMV DNAemia showed a strong cumulative effect on CXCL10 blood but not urine levels, an observation that is in line with recent findings that CXCL10 in blood but not in urine increased in KTRs with histologically verified HCMV infection 33. Of note, we previously demonstrated that intrapulmonal HCMV replication in lung transplant recipients caused a systemic rise of CXCL10 in blood, while in bronchoalveolar fluid it only increased when inflammatory airway obstruction simultaneously occurred 21. In the KTRs with HCMV DNAemia we investigated, the renal allograft might thus not have been the primary site of replication or inflammation.

Finally, it is noteworthy that among the T‐cell‐associated chemokines, we investigated, only CXCL10 and CCL8 significantly correlated with progression of BKPyV replication, while CCL20 and CXCL16 did not. Elevated CXCL16 concentrations were found in KTRs with hypertensive kidney injury, while increased CCL20 levels were detected in clinically manifest allograft rejection 48, 49. Interestingly, in vitro data demonstrated that renal tubular epithelial cells, when stimulated with IFN‐γ, strongly upregulate CXCL10 but not CCL20 50. Thus, due to the effect of virally induced Th1 cytokines, CXCL10 might predominate over CCL20 during BKPyV replication, indicating that different virus infections might indeed elicit different patterns of chemokine responses.

The lack of baseline samples and histological data for the whole cohort certainly pose limitations of this study. Nonetheless, in summary, the provided data demonstrate that CXCL10 in urine and blood already rises during early BKPyV replication, gradually increases in proportion with the extent of viral replication and correlates with the progression toward PVAN.

## Authorship

LW: designed the study, wrote the paper, performed the research and analyzed data. LH, YW and SWA: performed the research. RS and IH: participated in research design. GB and KH: participated in research design and wrote the paper.

## Funding

This work was supported by the Sigrid Jusélius Foundation, the Jane and Aatos Erkko Foundation, the Medical Society of Finland (FLS), and the Helsinki University Hospital Research and Education Fund. Lukas Weseslindtners research stay at the Department of Virology Department of Virology of the University of Helsinki was funded by the Austrian Science fund (Erwin Schrödinger fellowship J3962‐B30).

## Conflicts of interest

The authors declare no conflicts of interest.

## Supporting information


**Figure S1** **.** Association of CXCL10 with high level BKPyV DNAemia and BKPyV associated disease.

**Table S1** **.** Clinical information of study patients.

**Table S2** **.** Quantitative values of CXCL10 measurements.

**Appendix S1.** Material and Methods.
