# Supplementary material for: Longitudinal assessment of the CXCL10 blood and urine concentration in kidney transplant recipients with BK polyomavirus replication—a retrospective study
Source: Transpl Int. 2020 Feb 13;33(5):555–66. doi: 10.1111/tri.13584 (PMC7216881; doi:10.1111/tri.13584)
Supplement: Supplementary file 1 — Figure S1 . Association of CXCL10 with high level BKPyV DNAemia and BKPyV associated disease. [file TRI-33-555-s001.pdf]

Supplemental Figure 1: Association of CXCL10 with high level BKPyV DNAemia and BKPyV associated disease

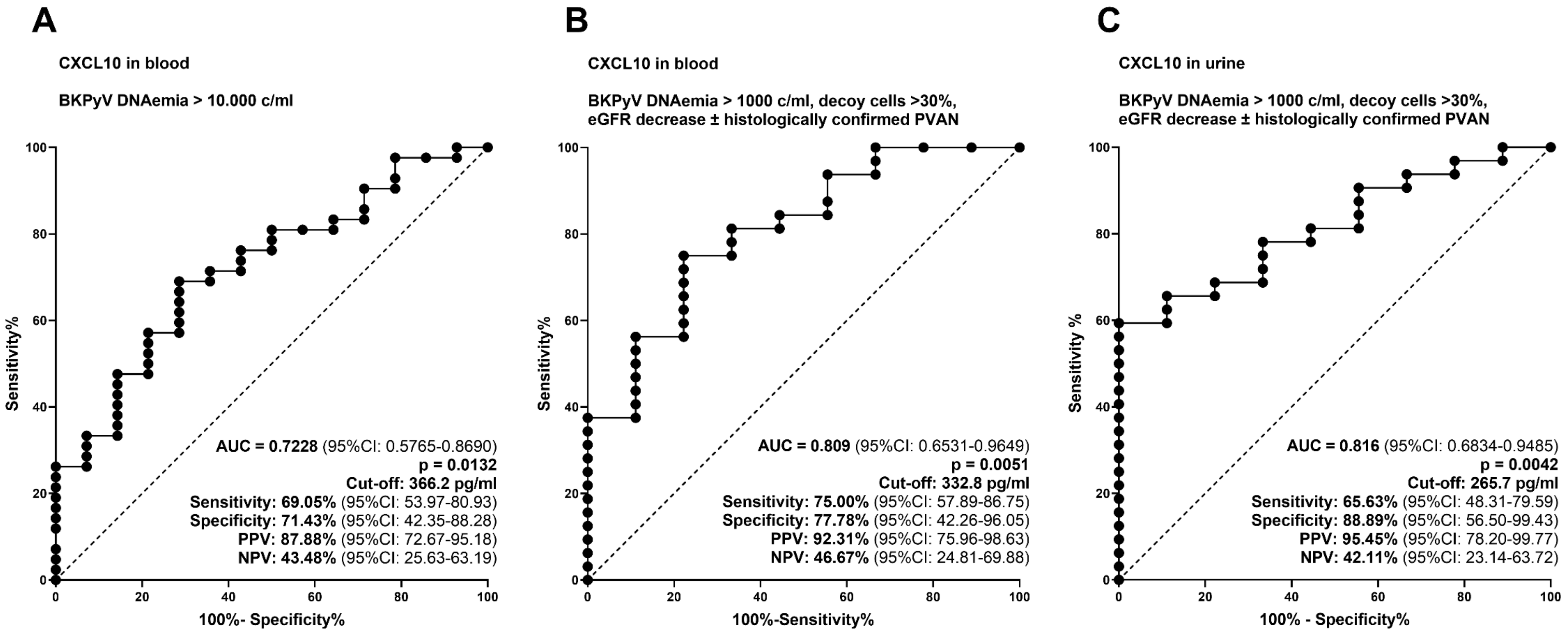

ROC analyses on the discriminatory ability of CXCL10 for (A) high-level BKPyV DNAemia >10.000 copies/ml presumptive for PVAN (CXCL10 in blood) and (B, C) for clinical manifestations and/or histological evidence of PVAN as defined in Table 2 (B: CXCL10 in blood; C: CXCL10 in urine).

(A) The CXCL10 blood concentration with an optimal cutoff of 366.2 pg/ml shows an AUC of 0.7228 and a PPV of 87.88% to differentiate between 42 KTRs with high-level BKPyV DNAemia (>10.000 copies/ml) and 14 KTRs with viral loads >1000 copies/ml but <10.000 copies/ml.

(B) CXCL-10 blood levels with an optimal cutoff of 332.8 pg/ml show an AUC of 0.809 and a PPV of 92.31% to differentiate between 32 BKPyV DNAemic KTRs with decoy cell levels >30%, concurrent decrease of renal function and/or histological evidence of PVAN and 9 KTRs with no significant decoy cell levels and no decrease of renal function (Table 2).

(C) For urinary CXCL-10 levels with an optimal cutoff of 265.7 pg/ml, the AUC is 0.816 with a positive predictive value of 95.45% for decoy cell levels >30%, a concurrent decrease of renal function and/or histological evidence of PVAN.
