# Supplementary material for: Longitudinal assessment of the CXCL10 blood and urine concentration in kidney transplant recipients with BK polyomavirus replication—a retrospective study
Source: Transpl Int. 2020 Feb 13;33(5):555–66. doi: 10.1111/tri.13584 (PMC7216881; doi:10.1111/tri.13584)
Supplement: Supplementary file 2 — Table S1 . Clinical information of study patients. [file TRI-33-555-s002.pdf]

Supplemental Table 1: Clinical information of study patients

patient groups	number of patients	Gender	age (mean, range; years)	primary diseases	TX type	donor				cold ischemia time (hrs.min, range)	delayed graft function (>=2 dialyses post-trans-plant)	HLA-mis-matches (A+B+DR, mean, range)	AB0- in-compati-bility +	HLA-in-compati-bility (PRA latest >40% or DSA) ++	duration of follow-up (median, range; days)	median time point of BKPyV DNAemia (day post-transplant, range)
						type	donation after circulatory death	age (mean, range; years)	gender							
**BKPyV DNAemia**	**56**	f: 20<br>m: 36	52,<br>19-75	hypertensive kidney disease (n=8), polycystic kidney disease (n=8), IgA nephropathy (n=5), Goodpasture syndrome (n=3), Alport syndrome (n=2), embranous nephropathy (n=2), diabetic nephropathy (n=2), pyelonephritis (n=2), membranous glomerulonephritis (n=2), juvenile sarcoidosis (n=1), ciclosporin induced nephropathy (n=1), membranoproliferative glomerulonephritis (n=1), nephrocalcinosis (n=1), multiple myeloma (n=1), rapidly progressive glomerulonephritis (n=1), mesangioproliferative glomerulonephritis (n=1), Wilms tumor & nephrectomy (n=1), membranous glomerulonephritis (n=1), lithium-induced nephropathy (n=1),vesicoureteral reflux (n=1), post-infectious glomerulonephritis (n=1), chronic renal insufficiency of unknown etiology (n=10)	K, 1st TX: n= 45<br>K, 1st ReTX: n=6<br>K, 2nd ReTX : n=2<br>K, 3rd ReTX: n=1<br>K, 4th ReTX: n=1<br><br>K & P,1st TX: n=1	dec.: n=45<br>liv.: n=11	2/56	55, 15-85	f: 33<br>m: 23	2.03-<br>31.00	9/56	3, 0-6	2/56	13/56	999,<br>193-2423	152,<br>40-577
**BKPyV + JCPyV DNAemia**	**13**	f: 3<br>m: 10	60,<br>29-74	hypertensive kidney disease (n=2), diabetic nephropathy (n=2), ciclosporin induced nephropathy (n=2), acute on chronic kidney failure (n=1), polycystic kidney disease (n=1), chronic pyelonephritis (n=1), rapidly progressive glomerulonephritis (n=1), post-infectious glomerulonephritis (n=1), chronic renal insufficiency of unknown etiology (n=2)	K, 1st TX: n=12<br>K, 4th ReTX: n=1	dec.: n=12<br>liv.: n=1	0/13	61, 21-85	f: 5<br>m: 8	1.15-<br>29.30	3/13	4, 2-6	0/13	3/13	714,<br>131-1769	122,<br>82-559
**BKPyV + HCMV DNAemia**	**16**	f: 7<br>m: 9	53,<br>32-74	polycystic kidney disease (n=3), diabetic nephropathy (n=3), hypertensive kidney disease (n=1), post-infectious glomerulonephritis (n=1), IgA nephropathy (n=1), medullary cystic kidney disease (n=1), vesicoureteral reflux (n=1), chronic renal insufficiency of unknown etiology (n=5)	K, 1stTX: n=16	dec.: n=14<br>liv.: n=2	0/16	54, 15-78	f: 11<br>m: 5	2.00-<br>24.00	4/16	3, 1-6	0/16	1/16	632,<br>177-1855	191,<br>103-714
**controls**<br><br>(no BKPyV, JCPyV and HCMV DNAemia	**10**	f: 5<br>m: 5	43,<br>19-70	diabetic nephropathy (n=1), medullary cystic kidney disease (n=1), membranous glomerulonephritis (n=1), mesangioproliferative glomerulonephritis (n=1), focal segmental glomerulosclerosis (n=1), multicystic dysplastic kidney (n=1), post-infectious glomerulonephritis (n=1), medullary cystic kidney disease (n=1), chronic renal insufficiency of unknown etiology (n=2)	K, 1st TX: n=8<br>K, 1st ReTX: n=1<br>K, 2nd ReTX: n=1	dec.: n=7<br>liv.: n=3	1/10	52, 23-84	f: 3<br>m: 7	1.30-<br>29.03	1/10	3, 0-5	0/10	1/10	744,<br>342-2539)	none
**difference among groups p-value**		0.54	0.08		0.81	0.50	0.52	0.45	0.14	0.97	0.72	0.19	0.70	0.39	0.15	0.14

+ KTRs of AB0-incompatible allografts received blood group antigen specific immunoapheresis, and KTRs with high AB antibody titers underwent additional induction therapy with anti-CD20 antibodies.

++ KTRs of HLA-incompatible allografts received anti-thymocyte globulin and IgG immunoapheresis to according to a local protocol.

Abbreviations: f: female, m: male, K: kidney, P: pancreas, dec.: deceased, liv.: living
