# Supplementary material for: Longitudinal assessment of the CXCL10 blood and urine concentration in kidney transplant recipients with BK polyomavirus replication—a retrospective study
Source: Transpl Int. 2020 Feb 13;33(5):555–66. doi: 10.1111/tri.13584 (PMC7216881; doi:10.1111/tri.13584)
Supplement: Supplementary file 3 — Table S2 . Quantitative values of CXCL10 measurements. [file TRI-33-555-s003.pdf]

Supplemental Table 2: Quantitative values of CXCL10 measurements

	baseline	BKPyV DNAuria, no DNAemia	low level BKPyV DNAemia (<1000c/ml)	BKPyV DNAemia (≥1000c/ml) decoy cells <20%	BKPyV DNAemia (≥1000c/ml), decoy cells >20% ± PVAN	BKPyV DNAemia (≥1000c/ml) rejection	BKPyV + JCPyV DNAemia (≥1000c/ml resp.) decoy cells >20% ± PVAN	BKPyV + HCMV DNAemia	BKPyV DNAemia (≥1000c/ml) decoy cells <5% no eGFR decrease	BKPyV DNAemia (≥1000c/ml) decoy cells >30% eGFR decrease	BKPyV DNAemia (≥10.000c/ml) decoy cells >30% PVAN	controls
**number of patients**	56						13	16	9	12	20	10
**number of samples**	16	33	21	31	47	10	13	16	9	12	20	10
**CXCL10 in blood (pg/ml)**												
**Minimum**	34.93	58.31	111.27	134.80	163.67	445.42	383.42	447.38	134.80	220.05	192.34	49.68
**25% Percentile**	73.79	119.00	144.81	191.78	343.80	499.82	432.09	568.73	186.73	323.02	306.00	57.10
**Median**	87.28	163.90	192.52	252.77	404.44	657.82	466.03	892.87	266.97	436.02	426.42	86.47
**75% Percentile**	118.30	224.70	207.01	312.79	532.80	966.54	573.97	1306.71	368.66	583.29	606.81	111.30
**Maximum**	177.80	340.70	378.98	462.27	807.41	1913.8	878.44	6263.81	462.27	708.19	878.44	119.20
**Mean**	93.17	175.73	191.53	260.85	429.67	812.79	521.96	1358.20	279.38	451.94	466.36	84.36
**95% CI of mean**	73.94-112.39	147.33-204.12	164.17-218.90	229.49-292.21	385.60-473.74	487.52-1138.06	430.33-613.59	590.29-2126.10	196.09-362.66	356.77-547.10	375.39-557.33	64.91-103.82
**CXCL10 in urine (pg/ml)**												
**Minimum**	7.80	7.80	7.80	7.80	40.63	116.42	153.81	15.15	54.93	157.49	77.460	7.80
**25% Percentile**	10.46	17.27	29.16	54.93	182.48	261.06	289.87	146.49	112.93	196.77	184.09	9.01
**Median**	18.44	45.83	61.68	116.62	381.26	482.49	422.15	384.59	168.09	345.42	412.97	12.82
**75% Percentile**	34.40	102.86	104.72	215.68	457.85	722.68	678.90	544.15	248.85	553.01	531.83	35.12
**Maximum**	75.75	160.94	171.11	332.00	788.08	1070.7	1258.95	678.67	289.86	803.70	1258.95	58.30
**Mean**	26.90	59.41	70.20	136.65	333.88	516.36	525.86	372.54	174.96	396.71	431.47	20.41
**95% CI of mean**	14.82-38.99	42.10-76.72	48.07-92.33	101.38-171.92	281.15-386.61	307.78-724.94	325.48-726.24	259.27-485.82	115.93-234.00	254.73-538.69	291.90-571.03	8.29-32.53

Abbreviations: CI: confidence interval, c/ml: copies/ml, BKPyV: BK Polyomavirus, JCPyV: JC Polyomavirus, HCMV: Human Cytomegalovirus, eGFR: estimated glomerular filtration rate, PVAN: Polyomavirus associated nephropathy, pg/ml: picograms/mililitre
