# Supplementary material for: Longitudinal assessment of the CXCL10 blood and urine concentration in kidney transplant recipients with BK polyomavirus replication—a retrospective study
Source: Transpl Int. 2020 Feb 13;33(5):555–66. doi: 10.1111/tri.13584 (PMC7216881; doi:10.1111/tri.13584)
Supplement: Supplementary file 4 — Appendix S1. Material and Methods. [file TRI-33-555-s004.pdf]

Supplemental Material and Methods

Immunosuppressive treatment of patients, sample acquisition and virological routine testing schedule

During the post-transplant surveillance 93 patients received triple immunosuppressive treatment with mycophenolate, corticosteroids and tacrolimus. One patient received mycophenolate, corticosteroids and cyclosporine A, and one patient mycophenolate, corticosteroids and belatacept. During the post-transplant follow-up virological routine testing consisted of PCR analyses for BKPyV and JCPyV DNA in urine and blood, collected pairwise on the same day of the post-transplant follow-up respectively, and HCMV DNA in blood. The protocol of sample acquisition required collection right after KTX and at least in 3 month intervals thereafter, with additional tests performed if BKPyV/JCPyV/HCMV associated disease was suspected clinically. Accordingly, a total of 1974 pairs of urine and blood samples were acquired from the 95 patients for quantitative BKPyV and JCPyV PCR (median sample pair number per patient: 15, range: 4-50; median test interval: 28 days, range: 7-182). After the immediate time point of virological routine testing for BKPyV and JCPyV, blood and urine samples were frozen at -20ºC. For the CXCL10 analyses of this study, samples were then thawed, and there had not been any prior thaw freeze cycles.

Quantitative BKPyV and JCPyv PCR

Quantitative BKPyV and JCPyV PCRs were performed according to a standardized in-house protocol. Quantitative assessment of HCMV DNA was performed by Cobas Amplicor HCMV Monitor-Test Kit on a COBAS Amplicor Analyzer (Roche Molecular Systems, Branchburg, NJ, USA).

For BKPyV and JCPyV quantitative PCRs, DNA was extracted from 200 µl plasma using the automated NucliSens EasyMag extractor according to the manufacturer’s instructions (Biomerieux, Marcy l'Etoile, France). BKPyV and JCPyV DNA quantification was performed

using real-time Taqman PCR with primers and probes located within the minor capsid protein VP3. Amplification was done in separate reactions using a forward primer (JC-BK-1 TGC TCC TCA ATG GAT GTT GC) and a fluorescence labelled probe (JC-BK-TqM CGG GAC TGT AAC ACC TGC TCT TGA AGC with fluorophore FAM at the 5'end and TAMRA at the 3'end) and either a BKPyV specific reverse primer (BKPyV reverse AGC TGC CCC TGG ACA CTC) or a JCPyV specific reverse primer (JCPyV reverse CAC GGG GTC CTT CCT TTC). For the PCR, 5µl of template DNA were added to the amplification mixture, using TaqMan universal PCR master mix (PE Applied Biosystems, Waltham, MA), yielding a total volume of 25 µl. Uniform cycling conditions were: 3 min at 50°C, 10 min at 95°C followed by 45 cycles of 95°C for 15 sec, 55°C and 72°C each for 30 sec. For quantification standard proficiency panels distributed by the Quality Control for Molecular Diagnostics (QCMD, Glasgow, UK) were used.

Quantification of CXCL10, CCL8, CXCL16 and CCL20

Quantification of CXCL10 levels in urine and blood was performed using a commercially available ELISA (BD OPTEIA Human IP-10 ELISA Set, Becton Dickinson Biosciences, San Diego, CA, USA) using a modified protocol described previously [30]. The lower limit of detection was 7.5 picograms (pg) per ml. Urine and blood samples were tested in duplicates, undiluted, in 10-fold and 100-fold dilutions.

CCL8, CCL20 and CXCL16 were quantified by LuminexTM xMAP technology according to the manufacturer´s recommendations (Bio-Plex Pro™ Human Chemokine Assays and Bio-Plex Pro™ Reagent Kits, Bio-Rad, Hercules, CA, USA). The limits of detection were 0.04 pg/ml for CCL8, 0.1 pg/ml for CCL20 and 0.1 pg/ml for CXCL16.
